# Glycosylation reduces the glycan-independent immunomodulatory effect of recombinant Orysata lectin in *Drosophila* S2 cells

**DOI:** 10.1038/s41598-021-97161-2

**Published:** 2021-09-09

**Authors:** Pengyu Chen, Kristof De Schutter, Sonia Serna, Simin Chen, Qun Yang, Niels-Christian Reichardt, Els J. M. Van Damme, Guy Smagghe

**Affiliations:** 1 Laboratory of Agrozoology, Department of Plants and Crops, Faculty of Bioscience Engineering, Ghent University, 9000 Ghent, Belgium; 2 Department of Biotechnology, Faculty of Bioscience Engineering, Ghent University, 9000 Ghent, Belgium; 3 Glycotechnology Lab, Center for Cooperative Research in Biomaterials (CIC biomaGUNE), Basque Research and Technology Alliance (BRTA), Paseo de Miramon 194, 20014 Donostia-San Sebastián, Spain; 4 CIBER-BBN, Paseo Miramón 182, 20009 San Sebastián, Spain; 5 Center for Advanced Light Microscopy, Ghent University, Ghent, Belgium

**Keywords:** Biotechnology, Physiology, Entomology

## Abstract

Several plant lectins, or carbohydrate-binding proteins, interact with glycan moieties on the surface of immune cells, thereby influencing the immune response of these cells. Orysata, a mannose-binding lectin from rice, has been reported to exert immunomodulatory activities on insect cells. While the natural lectin is non-glycosylated, recombinant Orysata produced in the yeast *Pichia pastoris* (YOry) is modified with a hyper-mannosylated N-glycan. Since it is unclear whether this glycosylation can affect the YOry activity, non-glycosylated rOrysata was produced in *Escherichia coli* (BOry). In a comparative analysis, both recombinant Orysata proteins were tested for their carbohydrate specificity on a glycan array, followed by the investigation of the carbohydrate-dependent agglutination of red blood cells (RBCs) and the carbohydrate-independent immune responses in *Drosophila melanogaster* S2 cells. Although YOry and BOry showed a similar carbohydrate-binding profiles, lower concentration of BOry were sufficient for the agglutination of RBCs and BOry induced stronger immune responses in S2 cells. The data are discussed in relation to different hypotheses explaining the weaker responses of glycosylated YOry. In conclusion, these observations contribute to the understanding how post-translational modification can affect protein function, and provide guidance in the selection of the proper expression system for the recombinant production of lectins.

## Introduction

During the process of protein glycosylation, proteins can be decorated with N- or O-linked glycans. With nearly half of the known proteins being glycosylated, this process is one of the most important post- and co-translational modifications. The attachment of these carbohydrate structures is essential for the proper structure and biological function of the glycosylated proteins and different glycoforms can modulate the activity of the proteins^1^. For example, core fucosylation of glycan moieties on the epidermal growth factor (EGF) is necessary for binding to the EGF receptor (EGFR), and deletion of the core fucose from IgG1 can increase its cytotoxicity^2^. In addition, it was observed that N-glycan structures can influence the immunomodulatory activities of glycoproteins possibly by the shielding of immunogenic protein domains. For example, recombinant hemagglutinin (HA), a carbohydrate-binding protein and major component of the spike protein of the avian influenza virus H5N1, can be decorated with different N-glycans depending on the protein expression system, and in general, complex N-glycan structures attenuate HA-glycan recognition and induce weaker immune responses^3,4^.

Lectins are a large group of proteins with carbohydrate binding properties. Because of their binding specificity, many natural lectins, purified from different sources such as plant, algae and fungi, have potential applications in fields such as biotechnology, medical research, and crop protection. For large scale production, recombinant production of these lectins has been a common practice, especially if the natural materials are low in lectin content^5,6^. However, due to differences in post translational modifications (PTMs), such as glycosylation, between hosts, it is important to select the appropriate protein expression system. Generally, prokaryotic expression systems conduct less PTMs than eukaryotic ones.

Orysata is a mannose-binding lectin from rice. The lectin was first purified from salt-stressed rice plants and was shown to induce carbohydrate-binding dependent agglutination of red blood cells at concentration of about 300 nM^7^. In addition, treatment of insect cells, including S2 cells (*Drosophila melanogaster*), GUTAW cells (*Helicoverpa zea*), High5 cells (*Trichoplusia ni*) and Bm5 cells (*Bombyx mori*), with recombinant Orysata (rOrysata), produced using the *Pichia pastoris* yeast system (YOry), caused carbohydrate-independent aggregation of the insect cells^8^ as well as transcriptional expression of antimicrobial peptides (AMPs)^9^. However, the secreted rOrysata produced in the yeast contains a significant proportion of YOry that is N-glycosylated and carries a hyper-mannosylated N-glycan moiety^6^.

Since glycosylation can affect protein folding, structure and activity^1,10,11^, we studied the effect of the yeast N-glycan on the activity of the glycosylated YOry in comparison with the *E. coli* produced non-glycosylated Orysata (BOry). The carbohydrate-binding specificity of both rOrysata was investigated and compared using a microarray containing 144 different glycan structures^12^. In addition, the ability of both rOrysata to induce carbohydrate-dependent agglutination of red blood cells and the carbohydrate-independent immune responses in *Drosophila melanogaster* S2 cells was analyzed. The observations on the performance of these two rOrysata can be beneficiary to biotechnologists and biologists for understanding how post-translational modifications, such as glycosylation, can affect protein function, and provide insight and guidance in the selection of the proper protein expression system for the recombinant production of lectins.

## Results

### Bacterial rOrysata (BOry) is non-glycosylated and can interact with the glycan on YOry

**Figure 1:**
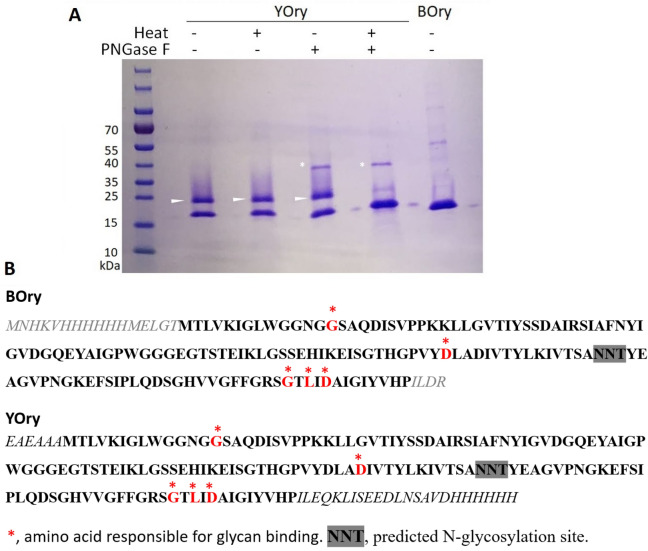
(**A**) SDS-PAGE of YOry and BOry. SDS-PAGE of BOry and YOry treated by heat and/or PNGase F. Orysata was inactivated by heating in 100 µl of PBS for 1 h at 98 °C. For the PNGaseF treatment, Orysata was diluted in PBS for 1 h at 98 °C, and then, 3 µl of PNGaseF (Promega) was added and incubated for 12 h at 37 °C. For the control, YOry was not heated but incubated with PNGase F. White arrows indicate the N-glycosylated YOry. * indicates the position of PNGase F on the gel. (**B**) Protein sequences for both BOry and YOry.

Recombinant Orysata was produced both in *E. coli* and *P. pastoris* and purified by Ni-affinity chromatography. Analysis by SDS-PAGE revealed that the rOrysata purified from bacteria (BOry) was present as a single band of the expected size (17.6 kDa) while the rOrysata produced in the yeast system displayed a double band (about 18 and 21 kDa) (Fig. 1A).

The double band represents a typical pattern for yeast produced glycosylated proteins with a single glycosylation site: the lower band represents the non-glycosylated YOry (18.5 kDa), while the upper band is the hyper-mannosylated glycoform (Man_(9–12)_GlcNAc_2_). This result agrees with the presence of a single N-glycosylation site identified in the Orysata protein sequence, NNT (Fig. 1B). A deglycosylation assay using PNGase F, revealed that native YOry could not be deglycosylated at 37 °C (Fig. 1A). After denaturation of the YOry by heat treatment at 98 °C, PNGase F was able to remove the N-glycan as seen by the single band on SDS-PAGE (Fig. 1A).

**Figure 2:**
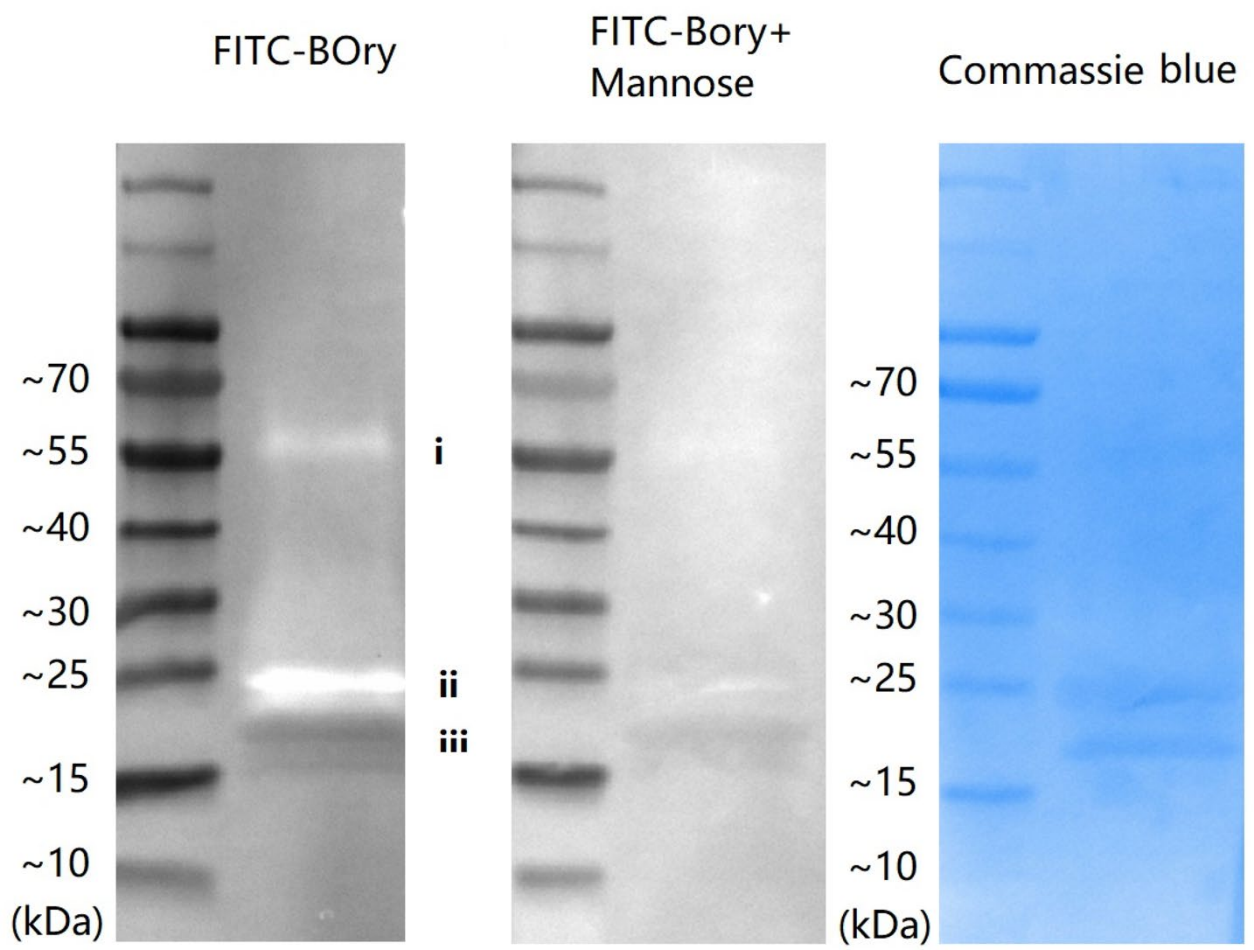
Glycan of YOry binding by BOry. The glycosylated (ii) and non-glycosylated (iii) form of YOry was separated by SDS-PAGE and blotted onto PVDF membrane. The blot was subsequently incubated with FITC-labeled BOry and fluorescent signals were detected using Image Lab. To confirm that BOry interaction with glycans is reversible, FITC-BOry was pre-incubated with 20 mM mannose in PBS, before being added to the YOry blot. The blot was finally stained by diluted Coomassie Blue. The band indicated with i is a possible aggregation of glycosylated YOry, and ii and iii indicate the glycosylated and non-glycosylated YOry respectively.

In a binding assay, it was shown that the N-glycan structure on YOry can be bound by BOry (Fig. 2). The glycosylated and non-glycosylated forms of YOry were separated on SDS-PAGE and blotted onto a membrane, subsequently the membrane was hybridized with FITC-labeled BOry. This revealed the interaction of FITC-BOry with the glycosylated form of YOry, while no binding to the non-glycosylated form could be observed. Preincubation of the FITC-BOry with mannose, abolished the binding to the glycosylated YOry (Fig. 2).

### Both rOrysata lectins have the same carbohydrate binding specificity

**Figure 3:**
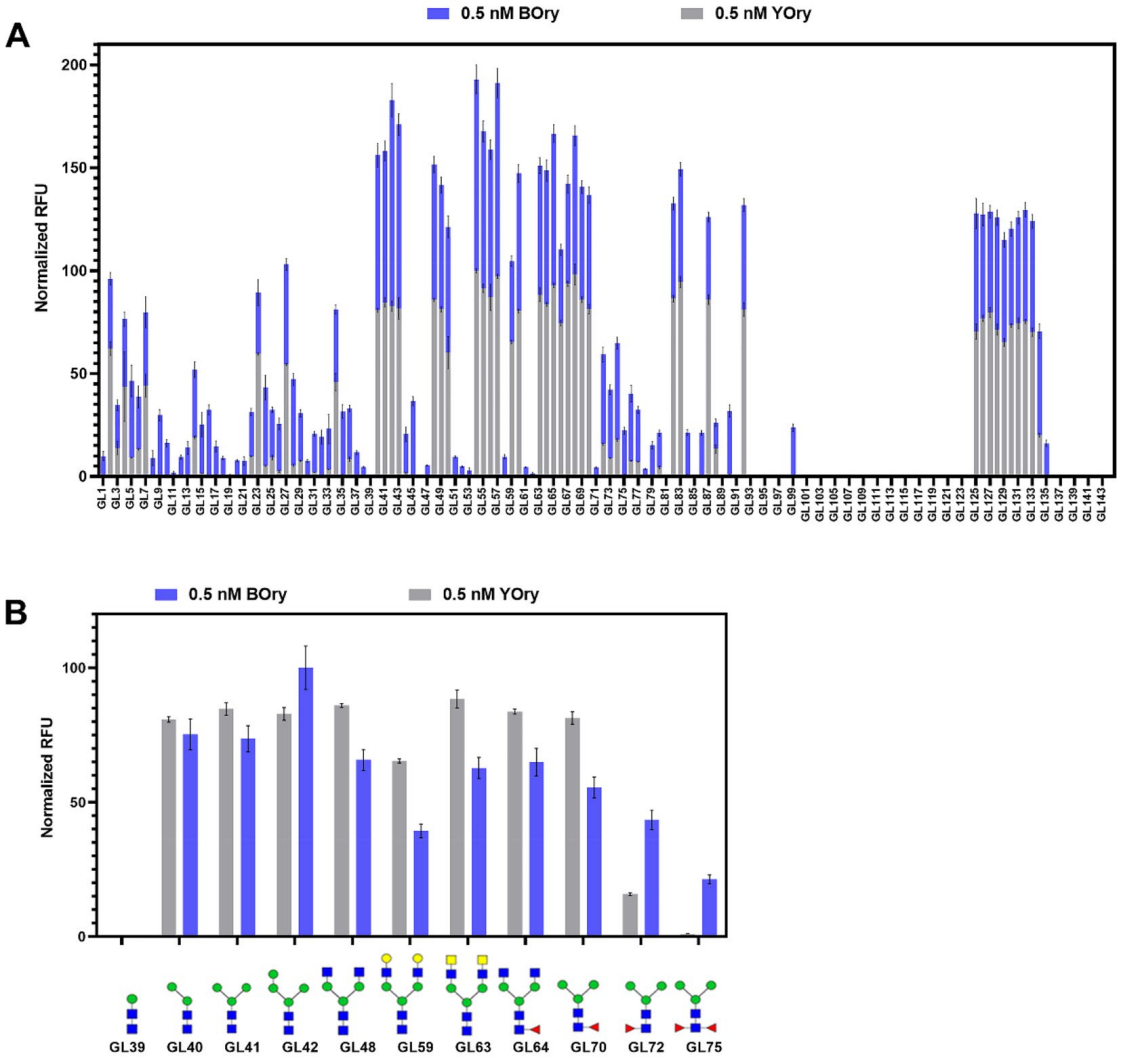
(**A**) Interaction of YOry and BOry with glycan array. Normalized RFU indicate the relative binding of BOry and YOry to different glycans (for glycan structures see Figure S1). (**B**) Comparison of YOry and BOry binding towards selected N-glycan structures discussed in the text.

To characterize the carbohydrate-binding specificity of both rOrysata lectins, a glycan array analysis was performed. The carbohydrate specificity of YOry had been previously analyzed on a glycan microarray (CFG glycan microarray version 4.2) composed of 511 glycan structures of mainly mammalian origin^6^. Recombinant YOry bound efficiently to various N-glycan type structures presented in the microarray. In the current experiment, a glycan microarray of 144 synthetic glycans with a large number of non-mammalian N-glycan structures (Fig. S1) was interrogated with YOry and BOry and reported us important additional binding details for both proteins. The collection of structures interrogated contained several paucimannose structures, N-glycans modified with core α-1,3-fucose, core β-1,2-xylose, LacdiNAc and fucosylated LacdiNAc (LDNF), epitopes commonly found in the glycome of insects, parasites, invertebrates, and plants. Glycan structures that were preferentially recognized, were identical for both YOry and BOry (Fig. 3A). A few additional glycan structures were bound by BOry, although their binding showed lower normalized RFU values (Fig. 3A). The paucimannose N-glycan GL40 was the smallest common structure efficiently bound by both recombinant lectins. An α-1,3-mannose residue linked to the β-mannose seems to be key structural element for efficient Orysata binding (Fig. 3B) while the α-1,6-mannose residue linked to the core β-mannose was not required. Overall, these results show a broad N-glycan binding specificity for rOrysata (Fig. 3B), efficiently recognizing paucimannose (GL40-41), high mannose (GL42-GL43) and complex N-glycans with terminal galactose (GL59), GlcNAc (GL48) and GalNAc (GL63). N-glycans functionalized with core α-1,6-fucose residues (GL64, GL70) showed important recognition towards rOrysata. But on the other hand, core α-1,3-fucose functionalized N-glycans, typically found on insect glycans reduced the interaction particularly to YOry (Fig. 3A).

YOry and BOry did not bind to other glycan classes present on our array (GL93-124, GL137-144) with the exception of the trimannose structure GL99 (Manα-1,2-Manα-1,2 Man), a fragment of the larger Man9 N-glycan that bound to BOry (Fig. 3A).

### BOry induces stronger agglutination than YOry

**Figure 4:**
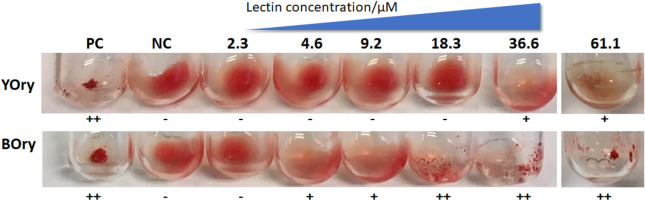
Agglutination activity of YOry and BOry. Trypsin-treated red blood cells (RBCs) from rabbit were used for the agglutination assays. After mixing the RBCs with different concentrations of the lectin, agglutination of the RBCs was scored. The lectin HHA was used as the positive control (PC), and PBS was used as the negative control (NC). +**+ **and + indicate a strong and good agglutination, respectively, while **–** indicates no agglutination.

To confirm lectin activity of the recombinant lectins, the ability of YOry and BOry to agglutinate rabbit red blood cells (RBC) was assayed. While both YOry and BOry can cause agglutination, the potency of the two rOrysata lectins differs (Fig. 4). For YOry, agglutination of the RBCs was observed starting at a concentration of 36.6 µM, while BOry can already cause agglutination at lower lectin concentrations, starting from 4.6 µM.

### Both YOry and BOry can induce immune responses in insect cells

The ability of rOrysata to induce a cellular immune response was assayed using *D. melanogaster* S2 cells. The lectins were screened for their potential to induce phosphatase-dependent, carbohydrate-independent aggregation of the cells, as previously reported for YOry^8^, and the ability to induce a morphological change in the cells, typical for the cellular immune response^13^.

**Figure 5:**
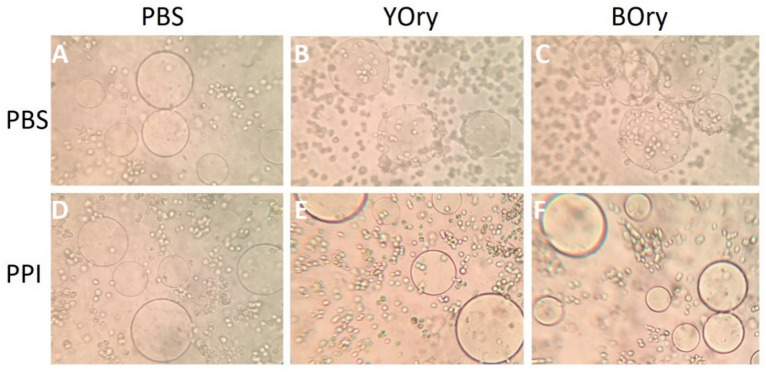
rOrysata-induced cell aggregation depends on phosphatase activity in S2 *Drosophila* cells. (**A**–**C**) In each well of the cell-repellent 96-wells plate, rOrysata-coated Ni–NTA agarose beads or PBS-treated blank beads were incubated with S2 cells for 40 min. (**D**–**F**) before adding the lectin-beads, cells were treated by 0.2× phosphatase inhibitors for 10 min. The formation of cell aggregation was recorded by cell phone camera attached to light microscope.

**Figure 6:**
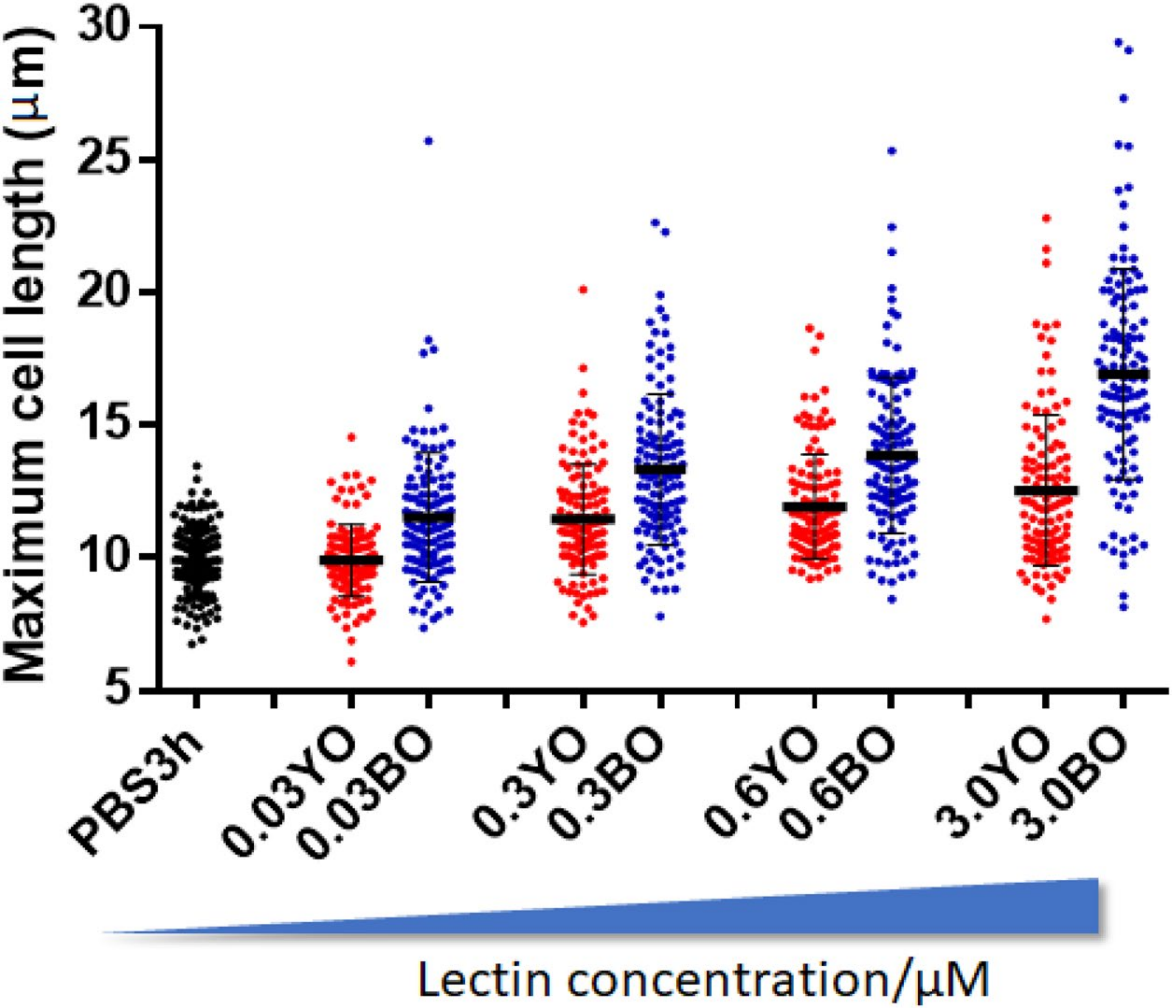
rOrysata induced cell spreading. 10^5^ cells were seeded overnight in a 24 well plate before addition of YOry (YO) or BOry (BO) to a final concentration of 3, 0.6, 0.3, and 0.03 µM. PBS was used as control. After an 3 h incubation, maximum cell length was measured using the ImageJ software. In total 120 cells were measured from 4 independent replicates. Statistical analysis results are shown in Table S1.

Incubation of the S2 cells with YOry- or BOry-coated agarose beads confirmed the ability of the rOrysata to induce aggregation of the cells (Fig. 5). While no cells were attached to the control beads after 40 min of incubation (Fig. 5A), aggregation of the cells to the coated beads was clearly observed (Fig. 5B,C). Preincubation of the S2 cells with phosphatase inhibitors (PPI) caused the inhibition of the aggregation for both YOry and BOry (Fig. 5D–F). During the early steps of cell aggregation, morphological changes, i.e. spreading and flattening of the cells, were observed for both YOry and BOry treatment. These morphological changes were quantified through the measurement of the maximum cell length (Fig. 6, Fig. S2). Under control conditions, the average cell length of the S2 cells was 10 µm. After 3 h incubation with 3 µM rOrysata the average maximum cell length increased to 13 µm and 17 µm for YOry and BOry, respectively (Fig. 6, Table S1). For YOry, significant difference in maximum cell length with the control was observed starting at a concentration of 0.3 µM, while BOry already induced a significant increase in cell length at concentrations of 0.03 µM.

**Figure 7:**
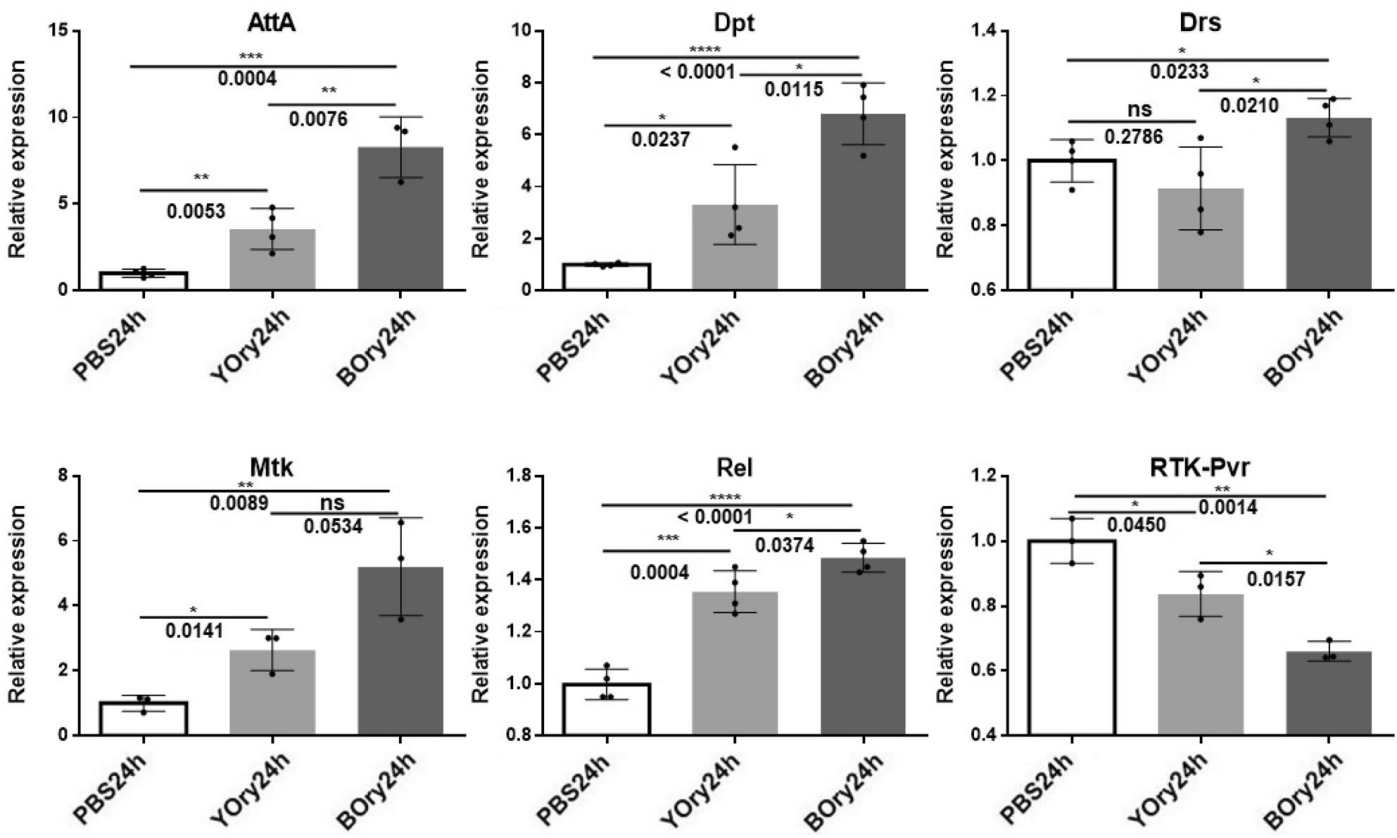
Relative expression of immune-related genes after YOry or BOry treatment of S2 *Drosophila* cells for 24 h. PBS treatment was used as a control. Data are analyzed by t-test, statistical significance is indicated by * and p values, n = 4.

Induction of the humoral response in S2 cells was screened by quantitative analysis of AMP transcription levels. Both YOry and BOry induce the transcriptional expression of the tested AMP genes, including Attacin A (AttA), Diptericin (Dpt), Drosomycin (Drs) and Metchnikowin (Mtk) (Fig. 7). Only for Drosomycin (Drs), the expression levels in the YOry treated cells are not significantly different from the control. Similar to previous results, BOry is a more potent inducer of the AMP gene expression compared to YOry (Fig. 7). In line with these observations, the transcript levels for the AMP regulator Relish (Rel, positive regulator) and a tyrosine kinase receptor Pvr (PDGF/VEGF homologue receptor, negative regulator), are up- and downregulated, respectively, by both rOrysata lectins. However, in agreement with the other results, this regulation is stronger in the cells treated with BOry compared to the cells treated with YOry.

## Discussion

Orysata is a jacalin-like lectin originated from rice. Yeast-produced recombinant Orysata (YOry), was found to possess immunomodulatory effects on *D. melanogaster* S2 cells. However, a portion of the YOry is modified with a yeast-type hyper-mannosylated N-glycan, while the wild type rice Orysata is non-glycosylated. As glycosylation has been shown to influence folding, structure and activity of the proteins they decorate^1,10,11^, in this study the importance of this N-glycan on the immunomodulatory activity of the lectin was analyzed by comparative study of the glycosylated yeast-produced Orysata (YOry) and the non-glycosylated bacterial-produced Orysata (BOry).

Both rOrysata lectins were shown to possess a similar carbohydrate-binding specificity, although some differences in binding intensity were observed. A previous study using frontal affinity chromatography (FAC) and glycan arrays indicated that Orysata bound to a broad spectrum high mannose N-glycans and also some complex-type glycans, while a lack of the α-1,3-mannose residue linked to the core β-mannose impaired Orysata binding^14^. The results of the current glycan array screening, including many invertebrate N-glycans, comes to similar conclusions.

While both YOry and BOry retained their ability to induce carbohydrate-dependent agglutination of RBCs, the minimum agglutination concentration was found to be tenfold lower for BOry compared to YOry. A similar observation was made when comparing the agglutination ability between native Orysata and the recombinant YOry. The minimum agglutination concentration of the native Orysata was found to be about 20-fold lower compared to YOry^6,7^. As the carbohydrate-binding specificity of both rOrysata lectins is similar, and no differences in targets of the lectins are expected, this difference in minimal agglutination concentration suggests that the presence of the glycosylation either causes a lower bio-availability of the carbohydrate-binding sites of YOry or impairs the Orysata dimer formation, leading to lower agglutination activity.

Similarly, analysis of the potential of the rOrysata to induce a cellular or humoral immune response in *D. melanogaster* S2 cells revealed that while both YOry and BOry cause the same response, the non-glycosylated BOry in general induces stronger effects. Immunomodulatory abilities of plant and fungal lectins have been well documented in vertebrate cells^15,16^, and recently, in insects similar effects were described^8,9^). The *Drosophila* S2 cells are hemocyte-like and both a cellular as well as a humoral immune response can be induced^13^. In the early steps of phagocytosis or encapsulation as part of the cellular response, hemocytes undergo morphological changes, the normally spherical cells become flattened and adhere to the substrate surface in a process called “cell spreading”. In this study, carbohydrate-independent aggregation and cell spreading was observed for both YOry and BOry. However, as for the carbohydrate-dependent agglutination, the minimal concentration to induce these effects were tenfold lower for BOry compared to YOry. Similarly, the induction of AMPs as part of the humoral response was observed after treatment of the S2 cells with both rOrysata, however, the transcript levels of these immune effectors were significantly higher when the cells were treated with BOry.

As both carbohydrate-dependent and carbohydrate-independent properties of Orysata are influenced by the glycosylation state of the lectin, it is suggested that the N-glycan affects not only the carbohydrate binding but also protein–protein interactions of the rOrysata. According to the structural model of Orysata^14,17^, the NNT N-glycosylation site is located on the C-terminal loop away from the carbohydrate binding site, thus the N-glycan should not impact the accessibility of the carbohydrate binding pocket. While the structural model of Orysata suggests the high mannose N-glycan is exposed^6^, in its native state, the N-glycan on YOry cannot be removed by PNGase F digest. This suggests that the N-glycan is protected, possibly through binding with the carbohydrate binding domain of another YOry because of its preference for mannose structures. This hypothesis is supported by the binding assay showing a carbohydrate-binding dependent interaction between BOry and the glycosylated YOry. This binding between different YOry molecules would decrease the bio-available carbohydrate binding sites and could also influence the dimer formation and protein–protein interactions. However, further research using different glycoforms of rOrysata would be needed to confirm or reject these hypotheses.

While the glycosylation status of proteins can impact protein function^3^, here no differences in carbohydrate-dependent and carbohydrate-independent activities were observed between glycosylated and non-glycosylated lectin. Interestingly, the effects caused by the non-glycosylated BOry were more outspoken compared to those caused by YOry treatment. Although the molecular or structural mechanism behind this phenomenon remains to be elucidated, these observations can offer a new perspective in explaining reduced activity of functional proteins, and for understanding how post-translational modifications, such as glycosylation, can affect protein function. In addition, this data warrants the careful selection of a proper protein expression system for the recombinant production of lectins and other proteins.

## Material and methods

### Protein purification, de-glycosylation, and fluorescence labeling

The coding sequence of Orysata (NCBI: XM_015766617) was amplified from the pPICZα::Orysata vector^6^ with primers KpnI-Ory and Ory-XbaI (Supplementary Table 1), containing the KpnI and XbaI restriction sites at their respective 5′ ends. The fragment was blunt cloned into the pJet vector (Thermo Fisher Scientific, Waltham, MA, USA) according to the manufacturer’s instructions and sequenced. Subsequently, the Orysata fragment was ligated into the pColdII (Takara bio, Kusatsu, Shiga, Japan) vector generating an in-frame fusion with an N-terminal poly-histidine tag. This recombinant vector, pColdII::Orysata, is transformed into competent *E. coli* strain BL21 cells. Transformed bacteria were selected on LB plates containing 100 µg/ml ampicillin.

For the bacterial produced Orysata (BOry), the recombinant BL21::pColdII::Orysata bacteria were grown to an OD600 of 0.5 and induced with 400 µM IPTG for 48 h. After harvesting the cells by centrifugation (7000*g* for 10 min at room temperature), the cells were resuspended in 20 mM of 1,3-Diaminopropane and homogenized by ultrasonication. The cell lysate was then centrifuged to collect the soluble protein fraction, which, after adjusting the pH to10, was applied to a QFF ion exchange column (DEAE Sepharose Fast Flow, Cytiva, Marlborough, MA, USA) and eluted by 0.1 M Tris–HCl pH 7.5 + 0.5 M NaCl. The pH of the eluted protein solution was adjusted to 7.5 prior to being applied to the Ni–NTA matrix column (Invitrogen, Carlsbad, CA, USA). After elution with 0.1 M Tris–HCl pH 7.5 + 250 mM imidazole, the buffer is exchanged to PBS using Amicon Ultra filters (Sigma-Aldrich, Saint Louis, MO, USA) with a molecular weight cut-off of 3 kDa. The protein sample was lyophilized and stored at − 20 °C until further use. For the production of the rOrysata in *P. pastoris* (YOry), the protocol was followed as described before^6^.

PNGase F was used to digest the N-glycan from both native YOry and heat denatured YOry. For the denaturing, 4 µg of YOry was diluted into PBS (pH 7.5) and heated at 98 °C for 1 h. 100 U of PNGase F (Promega, Madison, WI, USA) were added to the native and denatured samples and the samples were incubated for 12 h at 37 °C. As controls, native and denatured YOry without PNGase F treatment were included in the assay. Afterwards, all samples were analyzed by SDS-PAGE.

To verify the N-glycan of YOry can be bound by another Orysata, the glycosylated and non-glycosylated forms of YOry were first separated by SDS-PAGE, after which they were blotted to a membrane (FluoroTrans PVDF Transfer Membranes). Subsequently, the membranes were incubated with FITC labeled BOry for 20 min in the dark. After a gentle wash by PBS, FITC-fluorescence was detected using the Image Lab system (Bio-Rad, Hercules, CA, USA). To confirm the carbohydrate-lectin dependency, FITC-BOry was incubated with 20 mM mannose in PBS for 20 min, before being added to the YOry blot. The blots were finally stained by diluted Coomassie Blue and de-stained. BOry was labeled with FITC according to the product manual. Briefly, 2 mg of BOry was dissolved in 0.5 mL of 0.1 M sodium carbonate pH = 10. 50 µL of fresh 4 mg/mL fluorescein isothiocyanate (FITC, Sigma, Saint Louis, MO, USA) was added to the BOry solution in 10 steps of 5 µL each and, in between, the mixture was kept at 4 °C and mixed gently. After 8 h incubation while gently shaking at 4 °C in the dark, NH_4_Cl was added to a final concentration of 50 mM, and subsequently incubated for another 2 h at 4 °C. Finally, the free dye was removed by overnight dialysis in PBS pH = 7.4 at 4 °C.

### Glycan array analysis

The glycan array preparation and analysis was based on the method previously published^18^. Briefly, BOry was labeled with Alexa Fluor 555 NHS ester (Thermo Fischer Scientific) following manufacturer instructions and YOry binding was determined after incubation with rabbit-anti-His antibody (GenScript, Piscataway, NJ, USA) and Alexa Fluor-555 goat anti-rabbit IgG (Thermo Fischer Scientific). rOrysata (0.5 nM) was incubated in the microarray chip printed with 144 different glycans (Fig. S1). After washing, the fluorescence signal was analyzed on an Agilent G265BA microarray scanner system (Agilent Technologies, Santa Clara, CA, USA). Quantification of the signals was performed with ProScanArray Express (Perkin Elmer, Waltham, MA, USA) and Microsoft Excel software. The average of mean RFU values after background subtraction and standard deviation for four replicate spots was calculated. Average RFU values for each data set were normalized to the highest RFU value and represented as histograms employing Prism 6 software v6.07 (GraphPad, San Diego, CA, USA) (https://www.graphpad.com/).

### Agglutination assay

Rabbit red blood cells (RBCs) (purchased from Fiebig-Nährstofftechnik, Idstein, Germany) were prepared for agglutination assays as described before^6^. In brief, RBCs were rinsed twice with PBS before adding about 2 mg of trypsin and incubating for 10 min at 37 °C. Afterwards, the RBCs were rinsed three times to remove excessive trypsin and finally the RBCs were resuspended in PBS. For the lectin agglutination assays, 10 µl of 1 M ammonium sulfate was mixed with 10 µl of YOry or BOry lectin at different concentrations in a clean test tube. Subsequently, 30 µl of RBCs suspension was added and incubated for 30 min. The lectin *Hippeastrum hybrid* lectin (HHA) was added as a positive control (PC), and PBS was used as negative control (NC).

### Insect cell culture, aggregation assay, cell spreading and AMP expression

S2 cells from *Drosophila melanogaster*^19^ were purchased from the Bloomington *Drosophila* stock center (Indiana University, Bloomington, IN, USA) and are maintained under standard growing conditions in Sf-900 III SFM medium (Thermo Fisher Scientific) supplemented with 10% Gibco fetal bovine serum (FBS) (Thermo Fisher Scientific).

For the agarose beads-mediated aggregation assays, agarose beads were coated with rOrysata first. 10 µl of Ni–NTA agarose beads suspension were washed twice with PBS prior to adding 30 µg of YOry or BOry, PBS was supplemented to a final volume of 50 µl. After mixing at 4 °C for 1 h, excessive lectin was removed by washing the coated beads twice with PBS. Finally, the beads were equilibrated in 200 µl of PBS. In each well of a sterile CELLSTAR Cell-Repellent Surface 96-wells plate (Greiner Bio-One, Vilvoorde, Belgium), 60 µl of a *D. melanogaster* S2 cell suspension at a density of 10^6^ cells/ml in FBS-free medium was added. For the phosphatase inhibition assay, cells were treated with 20 µl of phosphatase inhibitor (PPI) for 10 min before adding 20 µl of Orysata-coated beads. For the PPI solution, one tablet of Pierce Phosphatase Inhibitor Mini Tablets (Thermo Fisher) was dissolved in 5 ml of PBS to make a stock solution for direct use.

To measure the cell spreading, 10^5^ cells were seeded in each well of a 24-wells plate in a total volume of 500 µL of cell culture medium supplemented with 10% FBS. After overnight incubation, 100 µL of YOry (YO) or BOry (BO) was added to a final concentration of 3, 0.6, 0.3 and 0.03 µM. PBS was used as control. After incubation for 3 h at 27 °C, cells were observed under the microscope and maximum cell length was measured using the image J software^20^ v2 (https://imagej.net/software/fiji/). In total 120 cells were measured from 4 independent replicates. Data was analyzed by one-way ANOVA with post-hoc Tukey HSD (Honestly Significant Difference) Test Calculator for comparing multiple treatments.

To quantify mRNA expression for AMPs, 100,000 cells were seeded in each well of a 24-wells plate (VWR, Leuven, Belgium) and incubated overnight as mentioned above. Afterwards, YOry or BOry were added to a final concentration of 3 µM. Cells were harvested after 24 h and total RNA was extracted using the RNeasy Mini Kit (Qiagen, Venlo, Netherlands) according to the manufacturer’s instructions. About 1 µg of purified RNA was used for cDNA synthesis with SuperScript IV Reverse Transcriptase (Invitrogen) in a 20 µL reaction system with polyT primers. After diluting the cDNA samples 20-fold, they were used as template for quantitative PCR (qPCR). For qPCR, every 20 µl of reaction system included 8.0 µl of cDNA template, 1.0 µl of primer F, 1.0 µl of primer R and 10 µl of GoTaq qPCR Master Mix (Promega). Reaction cycles are: 3 min at 95 °C, then 39 cycles of 10 s at 95 °C and 30 s at 55 °C. Relative expression was analyzed in qBase (Biogazelle, Gent, Belgium). The Ct values are normalized using two internal references, SdhA and RPL32. All primers are listed in Supplementary Table S2. Statistical differences were calculated in the qBase software.

## Supplementary Information

Supplementary Information 1.

Supplementary Information 2.
