# Supplementary material for: Glycosylation reduces the glycan-independent immunomodulatory effect of recombinant Orysata lectin in Drosophila S2 cells
Source: Sci Rep. 2021 Sep 9;11:17958. doi: 10.1038/s41598-021-97161-2 (PMC8429549; doi:10.1038/s41598-021-97161-2)
Supplement: Supplementary file 1 — Supplementary Information 1. [file 41598_2021_97161_MOESM1_ESM.docx]

**Supplementary information**

| **Table S1. Statistical analysis of maximum cell length** | | | | | |  |
| --- | --- | --- | --- | --- | --- | --- |
|  | PBS | 0.03YOry | 0.3YOry | 0.6YOry | 3.0YOry | |
| PBS |  | insignificant | ** p<0.01 | ** p<0.01 | ** p<0.01 | |
| 0.03BOry | ** p<0.01 | ** p<0.01 | insignificant | insignificant | * p<0.05 | |
| 0.3BOry | ** p<0.01 | ** p<0.01 | ** p<0.01 | ** p<0.01 | insignificant | |
| 0.6BOry | ** p<0.01 | ** p<0.01 | ** p<0.01 | ** p<0.01 | ** p<0.01 | |
| 3.0BOry | ** p<0.01 | ** p<0.01 | ** p<0.01 | ** p<0.01 | ** p<0.01 | |
|  | 0.3BOry | 0.6BOry | 3.0BOry |  |  | |
| 0.03BOry | ** p<0.01 | ** p<0.01 | ** p<0.01 |  |  | |
| 0.3BOry |  | insignificant | ** p<0.01 |  |  | |
| 0.6BOry |  |  | ** p<0.01 |  |  | |
|  | 0.3YOry | 0.6YOry | 3.0YOry |  |  | |
| 0.03YOry | ** p<0.01 | ** p<0.01 | ** p<0.01 |  |  | |
| 0.3YOry |  | insignificant | * p<0.05 |  |  | |
| 0.6YOry |  |  | insignificant |  |  | |

Maximum cell length data for 120 cells in 4 replicates treated with YOry or BOry were analyzed by One-way ANOVA with post-hoc Tukey HSD (Honestly Significant Difference) Test Calculator for comparing multiple treatments.

| **Table S2.** Primers used in this study | | |
| --- | --- | --- |
| Genes | Primer | Sequence |
| Orysata | KpnI-Ory | GGTACCATGACGCTGGTGAAGATT |
|  | Ory-XbaI | TCTAGAATAGGGTGGACGTAGAT |
| RPL32 | S2RPL32F | CCCAAGATCGTGAAGAAGCG |
|  | S2RPL32R | CGCACTCTGTTGTCGATACC |
| SdhA | S2SdhAF | AATGACCGTGTTCTTAGCGC |
|  | S2SdhAR | GCCCTGGATCTGATCTTCGA |
| Pvr | S2-qPvrF1 | ACACCGATCACATTGACCCC |
|  | S2-qPvrR1 | GTTGGCGGTTGCGTGTTATT |
| Drs | S2_Drs-F1 | CCAAGCTCCGTGAGAACCTT |
|  | S2_Drs-R1 | CAGGTCTCGTTGTCCCAGAC |
| Mtk | S2_Mtk-F1 | GCATCAATCAATTCCCGCCA |
|  | S2_Mtk-R1 | GCTCTGCCAGCACTGATGTA |
| AttA | S2AttAF1 | ACAAGCATCCTAATCGTGGC |
|  | S2AttAR1 | GGTCAGATCCAAACGAGCAT |
| Dpt | S2DptF1 | GCTGCGCAATCGCTTCTACT |
|  | S2DptR1 | TGGTGGAGTGGGCTTCATG |
| Rel | S2RelF1 | GCATGGAACACATGGATCGC |
|  | S2RelR1 | CTGATGGGAATGTGGGCTGT |


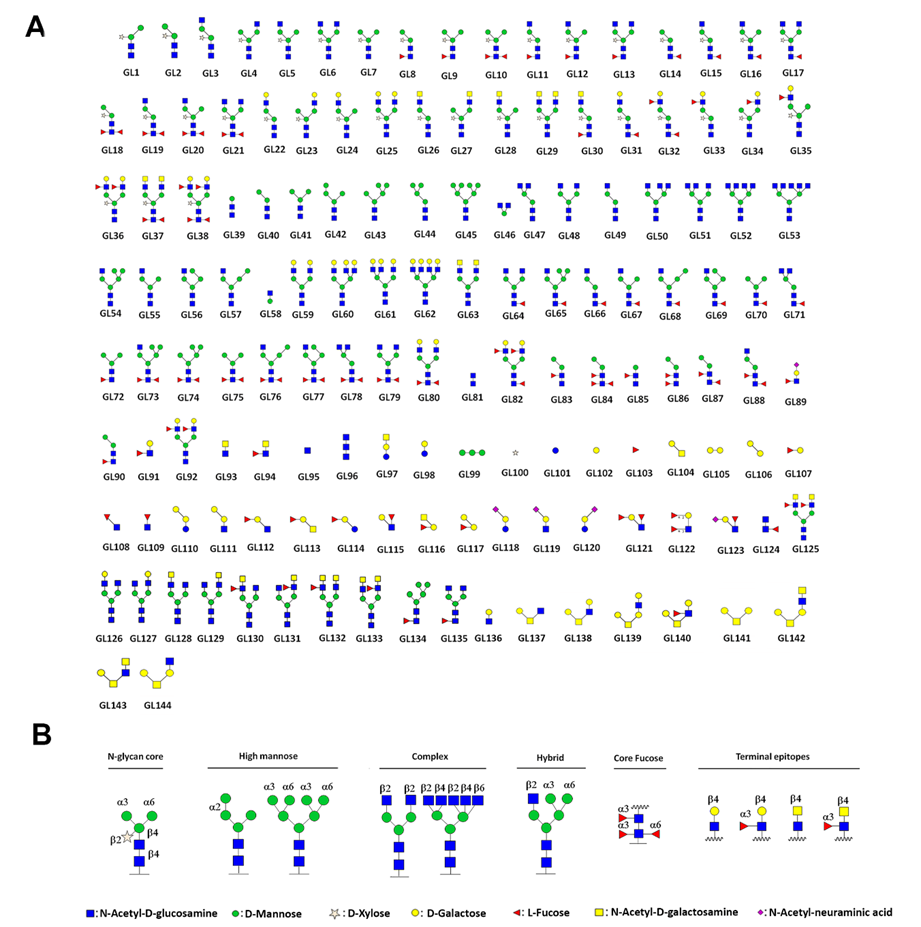


**Figure S1.** Glycan microarray employed in the study. A) Glycan structures included on the microarrays, B) Nature of the glycosidic linkages of the N-glycan structures on the microarrays. Figure generated with GlycoWorkBench v2.1 (Ceroni et al., 2008) (https://glycoworkbench.software.informer.com/2.1/).

Ceroni, A. et al. (2008) GlycoWorkbench: A Tool for the Computer-Assisted Annotation of Mass Spectra of Glycan. *J. Proteome Res.* 7, 1650-1659.


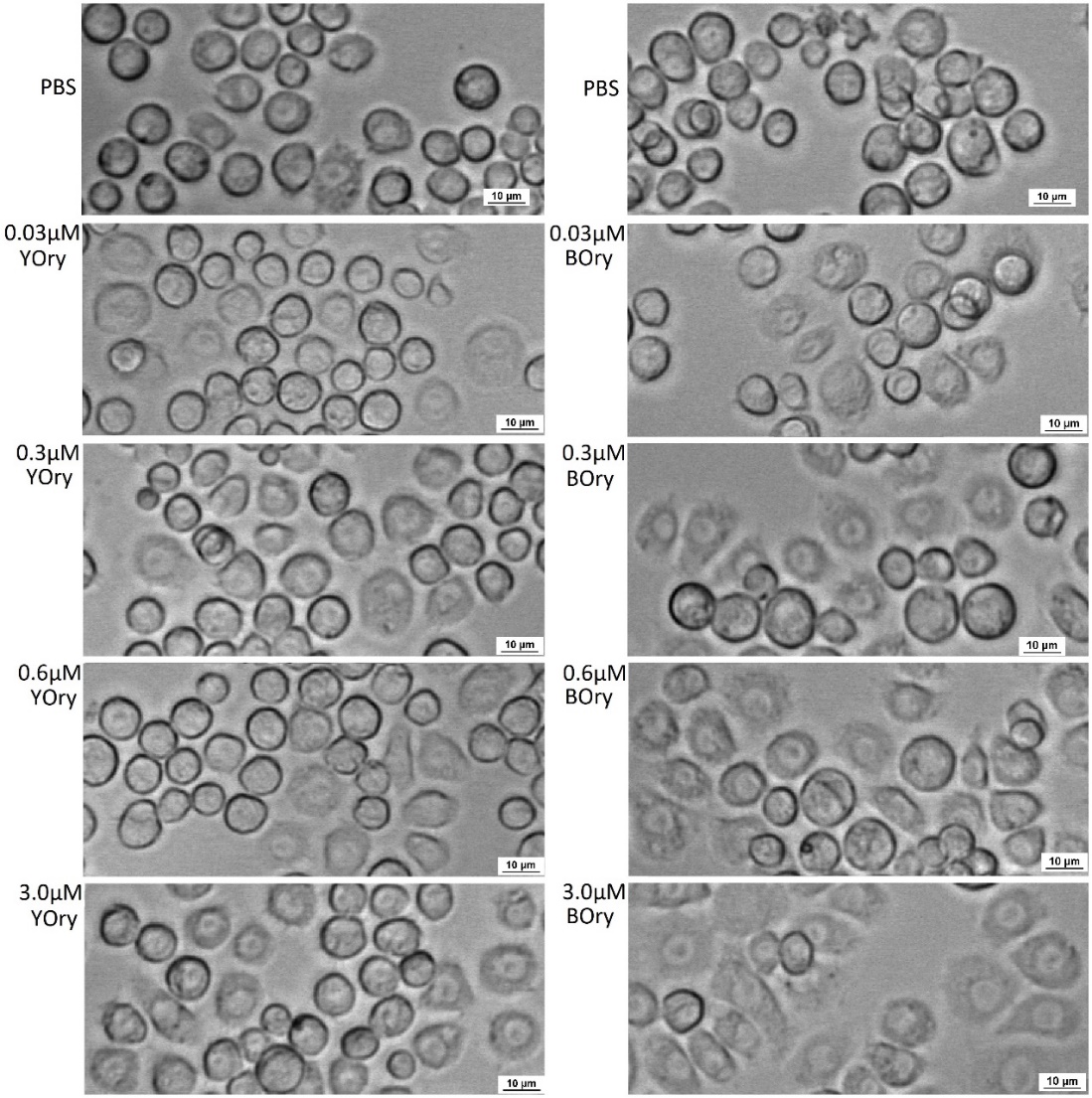


**Figure S2**. rOrysata induced cell spreading. S2 cells were incubated for 3h with different concentrations of YOry or BOry (3, 0.6, 0.3, and 0.03 µM). As control, cells were treated with PBS. Cell spreading was observed under the microscope. Size bar indicates 10 µm.
