# Supplementary figures and images for: Glycosylation reduces the glycan-independent immunomodulatory effect of recombinant Orysata lectin in Drosophila S2 cells
Source: Sci Rep. 2021 Sep 9;11:17958. doi: 10.1038/s41598-021-97161-2 (PMC8429549; doi:10.1038/s41598-021-97161-2)

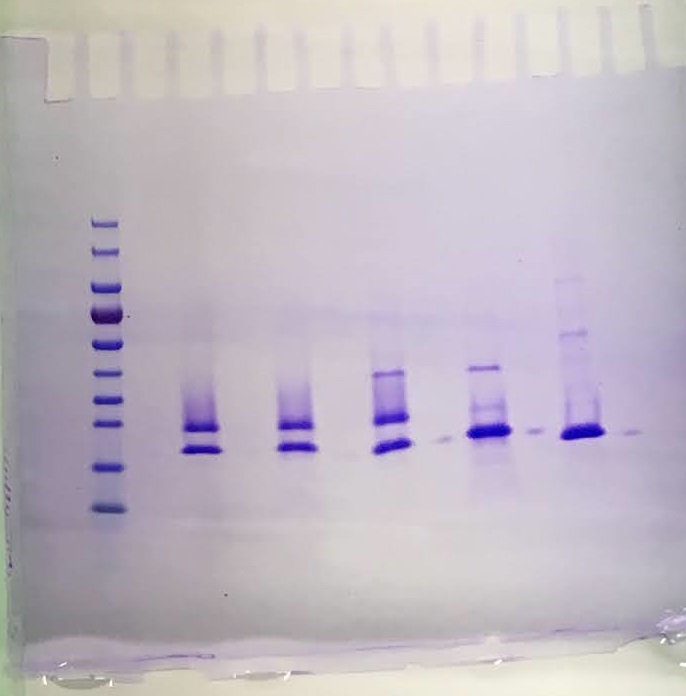

Supplement: Supplementary file 2 — Supplementary Information 2. [file 41598_2021_97161_MOESM2_ESM.jpg]
